# Women’s experiences with the self-management of hypertensive disorders of pregnancy: a descriptive phenomenological study

**DOI:** 10.1186/s12884-026-09007-2

**Published:** 2026-04-11

**Authors:** Shuang Hu, Danni Song, Yu Wang, Congshan Pu, Jiaai Xia, Chunjian Shan

**Affiliations:** 1 School of Nursing, Nanjing Medical University, Nanjing, China; 2 Department of Obstetrics, Women’s Hospital of Nanjing Medical University (Nanjing Women and Children’s Healthcare Hospital), Nanjing, China

**Keywords:** Pregnancy, Hypertension, Self-Management, Qualitative, Phenomenology, Maternal Health, China

## Abstract

**Background:**

Hypertensive disorders of pregnancy (HDP) is a significant contributing factor to maternal morbidity and mortality. Self-management plays a crucial role in women with HDP to delay disease progression and improve delivery outcomes. However, the lived experiences of self-management among women with HDP during pregnancy remain poorly understood. Therefore, the perspectives and experiences of managing HDP in women require special attention and exploration.

**Methods:**

The qualitative study with a descriptive phenomenological design was conducted. Purposive sampling was used, and face-to-face interviews were conducted with 19 women who had experienced HDP self-management. Data were analyzed following the 7 steps of Colaizzi. MAXQDA software (version 2020) was used for data management.

**Results:**

Four themes were identified: (1) the beginning of transformation, (2) the thorny path, (3) striving to overcome, and (4) external forces. The participants were found to be at different stages of HDP management and undergoing a process of adaptation.

**Conclusion:**

This study revealed that women go through a process of adjustment following a diagnosis of HDP as they learn to self-manage. This process is influenced not only by their personal efforts but also by the external environment and support systems.

**Supplementary Information:**

The online version contains supplementary material available at 10.1186/s12884-026-09007-2.

## Introduction

The World Health Organization (WHO) has established key targets aimed at “reducing maternal mortality” and “ending preventable deaths of newborns and children under 5 years of age” [1]. Hypertensive disorders of pregnancy (HDP) constitute one of the leading causes of maternal mortality globally [2], encompassing gestational hypertension, chronic hypertension, pre-eclampsia, eclampsia, and pre-eclampsia superimposed on chronic hypertension [3]. Globally, HDP affects 3–10% of pregnancies, accounting for approximately 14% of all pregnancy-related maternal deaths [4]. In China, the incidence of HDP ranges from 9.4 to 10.4%, accounting for 8% of such deaths [5, 6]. With the adjustment of reproductive policies, the increase in the age at first birth, and an earlier onset of chronic hypertension, the incidence of HDP in China has been increasing annually [7, 8]. HDP complicates approximately 8% to 10% of pregnancies [9], leading to the risk of adverse outcomes such as placental abruption, postpartum hemorrhage, and preterm birth in pregnant women and their fetuses [10]. Moreover, HDP increases the risk of a number of diseases, such as cardiovascular disease and chronic kidney disease, in both the pregnant women and their offspring in the future [11, 12].

HDP is a disease specific to pregnancy, and its detailed pathogenesis is unclear [13]. Early management of the diagnosed individuals can significantly mitigate disease progression, improve maternal and fetal prognosis, and reduce maternal and infant mortality [14–16]. However, most management is carried out by women, in addition to good diagnostic and instructional behavior by healthcare personnel. Women are the primary responsible people for the management of their diseases. Self-management refers to the process through which patients, under the guidance of healthcare personnel, master the knowledge and skills related to self-management and rely on themselves to solve problems [17]. Therefore, self-management of women with HDP is particularly important in order to delay disease progression and improve labor outcomes.

However, current research on HDP self-management primarily relies on quantitative tools and external observation. For example, Abheiden et al. [18] assessed aspirin adherence among pregnant women using a simplified medication adherence questionnaire, revealing an adherence rate of only 54.7%. Tucker et al.‘s [19] cross-sectional survey of 5,555 pregnant women revealed that only 49% engaged in self-monitoring of blood pressure. While such studies contribute valuable clinical and epidemiological aspects, they primarily employ quantitative research methods to answer the question “How many people do it?” without revealing the underlying “How is it experienced?”—that is, little is known about how women experience and understand HDP self-management. In recent years, qualitative research has begun to provide some insights into self-management among women with HDP. For example, Vinogradov et al. [20] explored the information needs of aspirin use during pregnancy in women with HDP, whereas Pealing et al. [21] used semi-structured interviews to understand the perceptions and experiences of self-monitoring of blood pressure in women with HDP. Despite these valuable insights, there remains a lack of in-depth qualitative research exploring the multifaceted experiences of self-management among women with HDP within the Chinese sociocultural context. Due to the high prevalence of HDP and its severe impact on pregnant women and fetuses, understanding patients’ subjective experiences is crucial for developing effective self-management interventions.

Therefore, this study employs a qualitative research approach, utilizing descriptive phenomenology to “return to the things themselves.” It delves into the lifeworld of women with HDP to capture their authentic experiences in self-management. This approach aims to drive intervention measures that genuinely respond to patients’ real-life circumstances and subjective needs, thereby enhancing the quality of healthcare delivery.

## Methods

### Study design

This study employs descriptive phenomenology, with its core objective being an in-depth exploration of the subjective experiences of women with HDP in self-management. This methodological choice is determined by the research question: capturing “the essence and meaning of self-management experiences.”The life-world approach based on Husserl’s [22] philosophy provides an irreplaceable philosophical foundation for this study. It acknowledges that patients’ subjective experiences themselves constitute a form of “truth”worthy of inquiry. By bracketing researchers’ preconceptions, personal biases, and clinical assumptions, this approach enables researchers to explore participants’ subjective experiences with greater openness and objectivity. It guides us to focus on “the relationship between participants and the world,” that is, analyzing how self-management behaviors are experienced within their specific historical, cultural, and social contexts. Since HDP women’s self-management experiences are rooted in both personal journeys and concrete backgrounds, this design enables a deeper exploration of individual perceptions. The consolidated criteria for reporting qualitative research (COREQ) guided this study to ensure rigor and scientificity.

### Study setting

This study was conducted at a Grade III Level A maternal and child health hospital in the provincial capital of Jiangsu Province, China—a core region of the Yangtze River Delta. By the end of 2024, the city’s total permanent resident population reached 9.577 million, with an urban population of 8.3607 million and an urbanization rate of 87.3%. As Jiangsu’s first Grade III Level A maternal and child health hospital, its obstetrics department is designated as a provincial clinical key specialty. The hospital maintains a comprehensive perinatal care and critical maternal rescue system. In recent years, it has provided over 400,000 outpatient visits annually, with inpatient services consistently ranking among the province’s top maternal and child health institutions. It leads provincial efforts in screening, diagnosis, management, and research of pregnancy-related complications, including HDP.

### Participants

This study was conducted at the obstetric outpatient clinic of Nanjing Women and Children’s Healthcare Hospital. Following ethics approval, researchers approached pregnant women with HDP during obstetric outpatient visits. Eligible patients were identified, provided with study details, and recruited if willing to participate. The primary interviewer (SH) had no prior relationship with any participant. All participants were recruited by other researchers during outpatient visits. The study employed a combination of purposive sampling and maximum variation sampling to ensure diversity in the sample across factors such as age, education level, and diagnostic type. The inclusion criteria were as follows: (a) age ≥ 18 years; (b) currently met the diagnostic criteria of “Diagnosis and Treatment of Hypertension and Preeclampsia in Pregnancy: A Clinical Practice Guideline in China (2020),” with a clear diagnosis of gestational hypertension, pre-eclampsia, pre-eclampsia superimposed on chronic hypertension, and chronic hypertension (our population includes participants both with and without a previous experience of HDP); (c) were able to speak and read Chinese; and (d) voluntarily participated and provided written informed consent. The exclusion criteria was (a) having a serious mental or psychiatric illness. This study determined sample size according to the principle of inductive thematic saturation [23]. This principle uses the absence of new codes or themes in data analysis as the criterion for saturation. The researcher observed no new codes emerging during the analysis of the 17th interview, prompting two additional interviews to confirm saturation. Recruitment concluded after the 19th interview. During the study period, 20 eligible potential participants were contacted. One individual refused to participate due to unwillingness to discuss their personal circumstances. Ultimately, 19 participants completed the interviews, resulting in a participation rate of 95%.

### Data collection

Data were collected from August 3, 2024, to September 27, 2024, through face-to-face semi-structured interviews. The interview guide was designed based on an examination of the literature and validated by obstetrics clinical nursing specialists. Two patients were pre-interviewed before the formal interview to confirm the logic and rationality of the interview guide. The final interview outline is shown in Table 1. The interviews were conducted by a female postgraduate nursing student (SH) with psychology knowledge and specialised interviewing skills. Prior to the study, the interviewer completed a comprehensive course on qualitative research methods and participated in multiple seminars on qualitative research. Before the interview, researchers will provide a detailed explanation of the study’s objectives, methodology, and significance, while repeatedly emphasizing the principles of anonymity and confidentiality. Following informed written consent, the participants were invited to share their real self-management experiences. The interview location was selected in a private, quiet room. Only one participant and one researcher (SH) were present during the interviews and there were no other interruptions. The entire interview process was audio-recorded, and the patient’s expression changes and body movements were recorded simultaneously. Each interview lasted 20–40 min. Each participant was interviewed on one occasion. Importantly, no patients were lost during the course of the interviews.

**Table 1:** Semi-structured interview questions

No.	Interview questions
1	How do you usually manage yourself?
2	What do you know about disease management?
3	What are the effects of HDP on your pregnancy and daily life? What are your thoughts?
4	What is the biggest difficulty you encounter in managing the disease? How do you feel when you encounter difficulties? How do you cope with it? What factors help you?

### Data analysis

Within 24 h of each interview, two researchers (YW and CP) transcribed the interview recordings verbatim into text. The transcribed text is validated to the patient. MAXQDA 2020 software was used to manage the data. In this qualitative study, data analysis was performed through the Colaizzi 7-step method [24]: (1) reading the collected interview data to develop a general understanding of the research object; (2) identifying and extracting statements relevant to the research problem; (3) constructing and coding the identified and extracted data; (4) summarising the coded ideas to find meaningful common concepts to form themes and theme groups; (5) providing a detailed description of the relationship between the themes and the research object; (6) forming the basic structure of the phenomenon; and (7) returning the resulting thematic structure to the object of study for validation. Two researchers (DS and CP) independently analysed the interview material to develop themes. Disagreements that arose were resolved through discussion within the study group. To ensure accuracy, both forward and back translations of the interview reports were independently completed by bilingual translators. To avoid potential bias, the back translators were unaware of the specific concepts these questions aimed to explore. Subsequently, the research team conducted a sentence-by-sentence comparison between the back-translated versions and the original transcripts, focusing on verifying the consistency of culturally specific expressions and contextual meanings. Discrepancies were discussed and resolved by the research group.

### Rigour of study

This study adhered to the principles of trustworthiness outlined by Lincoln and Guba [25]. These principles encompass credibility, dependability, transferability, and confirmability. To ensure credibility, only participants willing to openly share their experiences were included. After the interviews concluded, we returned the transcripts to participants and asked them to verify whether the content accurately reflected their original intentions. The researchers’ triangulation method also served as a strategy to ensure credibility. Two members independently analyzed the data, reaching consensus through discussion. If discrepancies arose, they were resolved through group discussion to achieve final agreement. Simultaneously, by integrating multi-source data from interviews, observations, and other methods, the research phenomenon was comprehensively presented. The study results were ultimately returned to participants for validation to ensure reliability. To enhance dependability, this study provides detailed descriptions of the methodology, conclusions, and ethical standards. To enhance the transferability of research findings, maximum variation sampling was employed to include respondents across diverse age groups, education levels, diagnostic categories, severity levels, parity, and gestational ages. Additionally, detailed documentation of the study setting and context, along with participant demographics, was maintained. These records enable other researchers to assess the relevance of the study’s findings to their own environments. To ensure confirmability, this study strictly adheres to the principles of reflexivity and bracketing.

### Reflexivity

This study emphasizes reflexivity and bracketing. By setting aside personal biases, preconceptions, and clinical assumptions, this approach enables researchers to collect and analyze data with a more open and objective mindset. To this end, the research group engaged in continuous self-reflection and discussion throughout the study. Prior to data collection, each member independently identified and documented their own assumptions and potential biases regarding self-management among pregnant women with HDP. This reflective exercise helped researchers recognize preconceptions and actively set them aside during data collection and analysis. The team held regular meetings to discuss and reflect on emerging data, enabling researchers to raise any perceived biases and support each other in maintaining objectivity.

### Ethical considerations

The study complied with the requirements of the Helsinki Declaration and was approved by the Medical Ethics Committee of Nanjing Women and Children’s Healthcare Hospital (2024KY-098). The original audio recordings and transcripts of all participants were maintained strictly confidential throughout the study and were used for this study only.

## Results

We interviewed a total of 19 women. The age of the participants ranged from 24 to 41 years. The gravida results revealed that 15 of the participants were primigravidae, whereas 4 were multiparous. Seven participants had received secondary education; twelve had achieved a tertiary education. The participants’ diagnoses and demographics are summarised in Table 2. This study extracted a total of 139 meaning units related to self-management experiences. By summarizing and condensing each meaning unit, 27 initial codes were formed. Subsequently, through multiple rounds of comparison and discussion, the research team clustered initial codes with similar meanings into more general categories. Ultimately, 12 sub-themes and 4 main themes were consolidated and refined. (Table 3)

**Table 2:** Participants’ Diagnoses and Demographics.(*n* = 19)

NO.	Age	education level	Diagnosis	Severity	No.of parity	Gestational age (in weeks +days)
1	30	Secondary	Chronic hypertension	Moderate	0	17 + 0
2	24	Tertiary	Chronic hypertension	Moderate	0	15 + 4
3	32	Secondary	Chronic hypertension	Moderate	0	32 + 4
4	32	Tertiary	Chronic hypertension	Moderate	0	25 + 2
5	33	Secondary	Chronic hypertension	Severe	0	24 + 4
6	29	Tertiary	Gestational hypertension	Moderate	0	27 + 0
7	29	Tertiary	Chronic hypertension	Moderate	0	37 + 2
8	27	Tertiary	Gestational hypertension	Moderate	1	34 + 2
9	35	Tertiary	Chronic hypertension	Moderate	1	32 + 5
10	28	Secondary	Chronic hypertension	Moderate	1	27 + 6
11	39	Secondary	Gestational hypertension	Moderate	2	26 + 0
12	29	Tertiary	Pre-eclampsia	Moderate	0	38 + 6
13	31	Tertiary	Pre-eclampsia	Severe	0	37 + 5
14	35	Tertiary	Pre-eclampsia	Severe	0	36 + 3
15	32	Tertiary	Pre-eclampsia	Severe	0	33 + 4
16	29	Tertiary	Gestational hypertension	Severe	0	36 + 6
17	41	Tertiary	Gestational hypertension	Moderate	0	38 + 5
18	39	Secondary	Pre-eclampsia superimposed on chronic hypertension	Severe	0	36 + 4
19	30	Secondary	Pre-eclampsia	Severe	0	35 + 3

**Table 3:** The themes

Themes	Sub-themes
The beginning of transformation	Intertwining of identities
	Relentlessness of disease
The thorny path	Cognitive limitations
	The difficulty of integrating cognitive and behavior
	Trapped in a vicious cycle
	Multiple uncertainties
Striving to overcome	Psychological adjustment and reshaping
	Summarizing and optimizing through practice
External forces	The power of role models
	Multifaceted support
	Conflicting perspectives
	Compromise and resignation

### The beginning of transformation

In this theme, the participants discussed how they initiated self-management. The intertwining of motherhood and patienthood and the relentlessness of the disease prompted participants to embark on a“journey of transformation.”

### Intertwining of identities

This preliminary phase of adjustment was accompanied by an intertwining of identity, i.e., the gradual transformation of the woman from an autonomous individual to a being who needed to shoulder the responsibilities of motherhood. The new roles and responsibilities led participants who had hypertension prior to pregnancy to begin to realising the importance of disease self-management. The participants described this shift as prompting them to pay more attention to their health status and lifestyle.

*“Before pregnancy*,* I did not get into the habit of monitoring my blood pressure because of my busy schedule. After pregnancy*,* I started to pay attention to blood pressure changes every day and gradually realised the importance of blood pressure management. I also started to pay attention to controlling my diet and maintaining a healthy lifestyle.” N9*

Some participants were faced with a sudden diagnosis of the disease during pregnancy, which was shocking and more bewildering than hypertension experienced before pregnancy. Nonetheless, the participants took on the burden of motherhood and patienthood status, controlling their blood pressure with medication and making positive lifestyle changes.

*“Since I was diagnosed with high blood pressure when I was pregnant*,* I have started taking medication and making positive lifestyle changes.” N1*

### Relentlessness of disease

Another powerful source of motivation is their desire to avoid complications and adverse experiences. After recognizing the seriousness of HDP, participants indicated they could understand the relationship between blood pressure outcomes, dietary adjustments, and self-monitoring, and were able to make positive changes.

*“I have learned that this disease is more dangerous for both adults and fetuses. Its management is largely self-reliant*,* especially in terms of dietary regulation and blood pressure monitoring.” N1*

Some participants had only mild HDP symptoms in previous pregnancies, whereas some women who had severe adverse experiences were prone to recalling past painful experiences and feelings and thus began to raise concerns about their blood pressure.

*“(During my first pregnancy)I did not know much about managing high blood pressure and did not pay as much attention to changes in blood pressure as I do now. It was not until just before the birth that my blood pressure rose to 170/190 and I was diagnosed with severe pre-eclampsia.” N9*.

Whether or not they have experienced pregnancy or similar health issues, women with prior experience may have a clearer understanding of the situation they face. They compare their current condition to their previous pregnancy.

*“I will monitor the edema regularly by pinching my legs. This baby is doing better than the last one*,* which had more severe edema.”N9*

### The thorny path

This theme highlights the contradictions and dilemmas faced by participants in self-management, as indicated by the following sub-themes: cognitive limitations, the difficulty of integrating cognition and behavior, becoming trapped in a vicious cycle, and multiple uncertainties.

### Cognitive limitations

Participants initially lacked essential awareness of HDP-related symptoms and risks, often unable to distinguish them from normal pregnancy changes. After diagnosis, they recognized past neglect of symptoms and expressed regret.

*“I developed edema very early on*,* starting with my feet… I just assumed it was normal.” N13*

*“Looking back now*,* I still feel I didn’t take it seriously enough… When my blood pressure was first flagged as abnormal*,* I thought it was no big deal.” N16*

Most participants failed to recognize the severity of HDP and did not understand the consequences of uncontrolled blood pressure.

*“It wasn’t until my blood pressure skyrocketed out of control that I realized hypertension is far more terrifying than hyperglycemia. I had no idea hypertension could be so serious before.” N17*

Additionally, participants held misconceptions about HDP self-management. For instance, Participant 13 believed self-management only required blood pressure monitoring, overlooking other critical aspects.

*“Blood pressure? Everyone just thinks you need to measure it.” N13*

### The difficulty of integrating cognition and behavior

Participants often struggle with cognitive-behavioral consistency. When confronting their existing unhealthy behaviors, they find deeply ingrained habits difficult to alter. The process of change frequently requires overcoming numerous obstacles, such as physiological cravings.

*“I didn’t know the harms of smoking*,* but I knew it was definitely bad. Yet I couldn’t control myself from smoking. Chewing gum or eating plums didn’t help. After meals or using the restroom*,* I’d crave a cigarette.” N3*

*“I drink about 7 cups of milk tea daily*,* usually managing 3 to 4 cups. I also love iced fruit tea… I know cold drinks and excessive milk tea aren’t good*,* but sometimes I just can’t control myself.” N7*

Faced with new disease management requirements, most participants recognize their benefits for disease prevention and control. Yet they remain caught in a constant tug-of-war between “striving to meet high management standards” and “being defeated by reality,” leading to a stalemate in disease management plans. One participant admitted that while choosing brown rice as their staple food, it sometimes proved difficult to stick to.

*“I try to choose brown rice as my staple food*,* but lately*,* for some reason*,* it seems increasingly hard to swallow.”N7*

Medication adherence is a critical component of HDP treatment, and ensuring regular medication intake is important. However, some participants admitted that the complex and cumbersome medication plans posed significant challenges. One participant stated:

*“The drugs need to be taken frequently.The process of taking medication frustrates me. ” N10*

### Trapped in a vicious cycle

The actual process of self-management is often described by participants as a “vicious cycle,” with participants explaining that they frequently find themselves trapped in a cycle that is difficult to escape. Most participants reported that sleep patterns and daily emotional states influence blood pressure monitoring results, while poor blood pressure readings further exacerbate the deterioration of daily emotional states and sleep patterns.

*“In early pregnancy*,* I found my blood pressure very difficult to control… This situation made me extremely anxious and uncomfortable*,* which caused my blood pressure to rise even higher*,* creating a vicious cycle.” N4*

*“Suboptimal sleep affects my daytime functioning and impacts my blood pressure. Poor blood pressure then leads to more sleep disturbances.” N9*

More challenging is that this “vicious cycle” is often difficult to break, subsequently affecting all aspects of self-management.

*“So many factors influence blood pressure—rest*,* diet*,* sleep… it’s affected by so many things. Look at me these past few days—my blood pressure is still unstable*,* and I’m not sleeping well either.” N12*

### Multiple uncertainties

During self-management, participants often feel helpless and disheartened when significant efforts yield no expected improvements. Some even begin questioning the effectiveness and value of self-management.

*“I’ve taken the medication*,* increased the dosage*,* adjusted my diet*,* and even walked more*,* but my blood pressure remains high. There’s nothing else I can do.” N15*

*“Measuring daily versus every few days doesn’t seem to make much difference. It feels meaningless. ”N11*

Facing poor blood pressure control, participants develop anticipatory anxiety, worrying about the timing and method of delivery, and fretting over potential pregnancy outcomes.

*“I’m worried my hypertension will worsen in the third trimester. It’s bad for both me and the baby.” N18*

*“I’m so anxious about how long this pregnancy will last.” N10*

### Striving to overcome

Participants actively adjust and adapt, seeking the most suitable self-management approach. This can be divided into two sub-themes: psychological adjustment and reshaping, and summarizing and optimizing through practice.

### Psychological adjustment and reshaping

Participants inevitably face challenges in self-management. However, most place these difficulties within a broader perspective, viewing them as worthwhile sacrifices for their children’s and their own health. By adjusting their mindset, they gradually restore balance and find pathways to overcome obstacles.

*“Although I initially felt annoyed by having to monitor my blood pressure multiple times daily*,* I’ve gradually adapted. After all*,* this isn’t just for my child’s health—it’s for my own long-term well-being.” N1*

Some participants also find comfort by comparing their circumstances to those of fellow patients facing more severe challenges.

*“Sometimes I hear about other pregnant women who take more medication than I do and seem to have more serious health issues. That’s when I comfort myself… Even though things are challenging*,* I’m managing well and don’t need to worry excessively.” N4*

Despite the hardships of disease management, participants re-examine past negative experiences, extracting positive meaning to fuel their progress, achieve personal growth, and even experience rebirth.

*“I’ve lived through this process. If I face similar illnesses in the future*,* I’ll pay closer attention to my physical condition—adjusting my diet*,* exercising*,* and getting regular check-ups.” N12*

*“I feel like I’ve been reborn.” N19*

### Summarizing and optimizing through practice

“Persistence”emerged as a core experience frequently mentioned by participants throughout their disease management journey. It proved to be the secret to implementing management strategies when confronting tedious and recurring challenges.

*“I walk every day without fail*,* regardless of the weather—rain or shine.” N1*

*“My advice is to strengthen management. For example*,* consistently measure blood pressure regularly.” N5*

The disease management content is similar, but participants actively explore and build personalized pathways. Some leverage various tools as aids to enhance management, making vague bodily sensations more intuitive and quantifiable. Others integrate disease management into daily rhythms, breaking down barriers between health management and everyday life.

*“I’ve consistently monitored fetal movements using an app.” N7*

*“I typically use mealtimes as reference points for blood pressure monitoring*,* developing the habit of checking my blood pressure before and after every meal.” N6*

### External forces

External influences can be both positive and negative. These influences are crucial for self-management. The topic is primarily divided into the power of role models, multifaceted support, conflicting perspectives, compromise and resignation.

### The power of role models

The health perspectives and behaviors demonstrated by family members and fellow patients serve as reference points for participants to perceive disease management approaches and significance.

*“My grandparents and father have chronic illnesses at home. My mother is responsible for administering their insulin and monitoring their blood sugar. Our family places great emphasis on health*,* which has also made me more attentive to managing my own condition.” N17*

*“When I have questions*,* I consult fellow patients in our WeChat group about their disease management experiences.” N10*

### Multifaceted support

During disease management, participants voluntarily relinquished some autonomy, while family members and healthcare providers intervened in care through various means, offering robust support. Family members provided assistance through practical care such as medication reminders and coordinating balanced diets, while healthcare professionals contributed through professional management knowledge and skillful guidance. Consequently, participants felt their health was prioritized, experiencing warmth and support. Simultaneously, this support alleviated the pressure of self-management, bringing a sense of ease.

*“Overall*,* the support and care I need—whether at the hospital or at home—has been fully provided*,* allowing me to manage my pregnancy health relatively easily.” N6*

Additionally, some participants felt understanding from colleagues and accommodating attitudes from supervisors. Flexible work hours, adjusted tasks, and acceptance of physical discomfort offered significant support, enabling participants to better focus on health management.

*“During early pregnancy*,* physical discomfort was quite pronounced*,* often making it difficult for me to work efficiently… Sometimes I communicated with my boss*,* requesting a few days off or informing him that on certain days I could only handle messages via my phone. My boss is very kind and understanding. He agreed to all these requests.” N4*

### Conflicting perspectives

In self-management within life contexts, external support is not merely a one-way exchange of assistance. Disease management involving family members and healthcare providers also carries implicit conflicts. For instance, self-management tasks requiring family assistance—such as meal preparation—may necessitate “warning education” to ensure certain family members take these responsibilities seriously and adhere to them. Participant 4 mentioned that her mother-in-law needed to be “scared” into taking management seriously, revealing a family dynamic of “persuasion-monitoring-warning.”

*“I make my family minimize salt use. My husband constantly reminds my mother-in-law to use less salt—just this much (gesturing with his hand). She’s the type who only takes things seriously when threatened*,* so he keeps warning her about the consequences if she doesn’t pay attention.” N4*.

Despite the extensive professional assistance provided by medical institutions, a complex and dynamic process of conflict and negotiation persists between medical authority and individual autonomy. While relying on external authority, participants maintain their agency through internal perception and reflection.

*“When the doctor advised me to stop taking my medication*,* I was utterly astonished and filled with questions—why would I need to stop? … I require long-term blood pressure medication*,* and at first*,* I simply dared not abruptly discontinue it. Later*,* I thought*,* since the doctor recommended stopping*,* I should follow medical advice.” N9*.

### Compromise and resignation

When disease management requirements conflict with family lifestyles, participants proactively adapt. They balance personal health responsibilities with family harmony by moderately distancing themselves from unhealthy habits without altering household routines.

*“We eat separately. I cook my own meals*,* freeze them*,* and steam them when it’s time to eat. They don’t like eating with me anyway.” N5*

When compromise fails to meet disease management requirements, resignation sets in.

*“My husband often comes home very late at night… Though I’ve asked him to return earlier*,* sometimes he gets caught up in his activities and loses track of time*,* only coming back late. This usually pushes our bedtime past eleven o’clock.” N5*

In workplace settings, participants’ self-management behaviors face new compromises as they adapt to work rhythms and schedule changes.

*“If I’m working on weekdays*,* I can only try to squeeze in time to complete these monitoring tasks*,* making it difficult to be as prompt as needed.” N6*

Participant 7 described facing unfair treatment in the workplace—layoffs—which forced pregnant women to endure not only personal management challenges but also occupational exclusion based on stereotypes at work. This led to multiple reactions involving stress, emotions, and physical health.

*“I was fired by the company because of my pregnancy. I had many conflicts with the company. My emotions fluctuated significantly*,* and the pressure became overwhelming.” N7*

## Discussion

This exploratory study focused on gaining a deeper understanding of women’s experiences in the self-management of HDP. The key themes that emerged evidenced the complexity and multi-layered nature of the self-management experience. Women experienced a process of adjustment as they embraced and undertook self-management tasks for HDP.

All participants in this study have the dual role of mother and patient with HDP, which motivated them to undertake self-management efforts primarily rooted in the desire to maximize the fetus’s health [26]. Yet, many struggle to understand the value of self-management. Their commitment to the maternal role manifested in various ways: eating disliked foods, self-monitoring blood pressure, taking medication on time, and attempting to fulfill exercise requirements. This aligns with findings by Newson [27], which linked fetal health and maternal bonds to greater pregnancy investment and adoption of health-promoting behaviors. This phenomenon was echoed in other studies of the experiences of women with HDP [28].

Although the participants had some knowledge about self-management of HDP, most still reported some cognitive misconceptions. For instance, similar to respondents in prior qualitative research, women in this study mentioned that symptoms of HDP are diffuse and difficult to differentiate from normal pregnancy symptoms [29]. Moreover, women with HDP generally perceive glucose management as more important than blood pressure management and neglect blood pressure management. This may be attributed to misconceptions about the disease and self-management, which have the potential to influence the outcome of pregnancy. It is therefore crucial to promote a correct understanding of the disease to effect positive behavioral changes [30].

Some participants also reported difficulty resisting the immediate gratification of current undesirable behaviors. Although they are aware of the potential negative impact of these behaviors on the future, they are torn between the long-term goals to restrain behavior and the immediate impulses that promise hedonic fulfillment [31]. A strong preference for immediate rewards and a lack of capacity for delayed gratification make it difficult for women to follow healthy self-management behaviors [32]. This is consistent with a study of chronic disease research [33]. This explains why some women who have recognized the importance of behavioral change still struggle to implement it.

The study also identified an aspect that has been less frequently addressed in previous studies, namely the “vicious circle” of self-management. Various aspects of self-management interact with each other [34], and the influence of one aspect can easily lead to the “loss of control” of other aspects, which together can exacerbate the instability of blood pressure. Moreover, uncertainty is one of the main factors affecting adherence to self-management [35]. Once participants did not feel significantly better, they may become skeptical about the effectiveness of management efforts, leading to the onset of uncertainty associated with the self-management of their illness. The women with HDP in this study are not only skeptical about the effectiveness and significance of self-management but also uncertain about the illness and future, which is consistent with other studies of chronic condition contexts [35].

In our study, sensitive support provided by family and healthcare professionals largely facilitated adaptation to HDP self-management. The importance of family support in facilitating women’s self-monitoring has also been demonstrated in previous studies [28]. Husbands or partners were the primary source of support, followed by her family, with particular sources of support being the participant’s mother-in-law and mother. In traditional Chinese culture, these elder women are viewed as experienced caregivers, and their care and support are recognized as a conventional duty and obligation [36]. These individuals encourage participants to complete daily HDP self-management tasks and help with more difficult aspects. However, at times, this care rooted in traditional roles and experiences, though well-intentioned, often conflicts with participants’ self-management practices informed by modern medical knowledge. Despite this, participants strive to strike a balance between maintaining family harmony and safeguarding their own health. Inadequate support can also have a negative effect on women’s adherence to self-management principles. Another critical yet often lacking form of support came from the workplace. Some participants faced not only intense work during pregnancy but also discrimination and stigma associated with their work. Protective measures in the workplace during pregnancy are essential to enable participants to have sufficient time for self-management. Reasonable support and understanding help alleviate the stress and promote emotional well-being, which in turn facilitates sustained self-management.

### Strengths and limitations

The findings of this study must be interpreted in light of its limitations. First, generalizations in phenomenological research are expressed as the essence of the phenomenon. The accuracy of the essence description requires verification in the specific context of its intended application. This study was conducted solely in a single institution in Nanjing, and the participants were all from urban settings with relatively adequate medical resources. This, to some extent, limits the external validity of the findings. Caution is warranted when attempting to directly generalize the results to rural areas, resource‑constrained regions, or non‑Chinese cultural contexts. Second, the study participants did not include women in their first trimester, as most women are not registered at their local hospital until 12 weeks of gestation and undergo a comprehensive prenatal check-up. Third, self-management is a developmental process that unfolds over time, necessitating a longitudinal study to thoroughly examine the evolution and changes in self-management experiences.

Despite these limitations, our study has important strengths. The selected population addressed four types of HDP to increase the scope of the problem identified. Second, the researchers were able to make in-depth contact with the participants to obtain descriptive information about the self-management of HDP. Additionally, the study followed the Lincoln and Guba principles of trustworthiness to help ensure the reliability and validity of the research process and results.

### Implications and recommendations

This study provides novel information for nurses, midwives, obstetricians, educators and policy makers to facilitate an improved understanding of the experiences of self-management from the perspective of women with HDP. Second, the integration of telemedicine, mobile healthcare, and smart monitoring devices into antenatal care [37] has the potential to enhance self-management in women with HDP by providing timely medical information and assisting in the management of the disease in daily life. However, the acceptability and precise implementation of these methods remain to be elucidated, and further studies are warranted to ascertain their efficacy in this population.

Finally, the importance of involving family members should be emphasised. This helps to create a positive family environment, from the provision of practical help to emotional support, all of which are essential for the self-management of women with HDP. It is also sad that some women in our study described negative experiences they had encountered during their working lives. The WHO and the International Labour Organization emphasise that pregnancy and childbirth can be vulnerable times for working women and their families, and that maternity protection should be provided for women workers. Ensuring that pregnant women receive adequate protection and support at work promotes fairer and healthier labour relations. Especially for pregnant women who remain in the workplace, it is crucial to provide a safe, healthy and respectful work environment.

## Conclusions

This study revealed that women go through a process of adjustment following a diagnosis of HDP as they learn to self-manage. This process is largely facilitated by women’s interest in maximising fetal health, which may make them receptive to interventions to improve HDP.

## Supplementary Information

Supplementary Material 1.

## Data Availability

No datasets were generated or analysed during the current study.

## Abbreviations

HDP: Hypertensive disorders of pregnancy
WHO: World Health Organization
